# 2-Hydr­oxy-*N*′-[(1*E*,2*E*)-3-phenyl­prop-2-enyl­idene]benzohydrazide

**DOI:** 10.1107/S1600536808028481

**Published:** 2008-09-13

**Authors:** Ning-Ning Ji, Zhi-Qiang Shi

**Affiliations:** a Department of Chemistry, Taishan University, 271021 Taian, Shandong, People’s Republic of China; b Department of Materials Science and Chemical Engineering, Taishan University, 271021 Taian, Shandong, People’s Republic of China

## Abstract

In mol­ecule of the title compound, C_16_H_14_N_2_O_2_, the two aromatic rings form a dihedral angle of 6.93 (3)° and an intramolecular N—H⋯O hydrogen bond occurs. In the crystal structure, inter­molecular O—H⋯O hydrogen bonds link the mol­ecules into zigzag chains running in the [10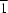] direction.

## Related literature

For the coordination chemistry of Schiff bases, see: Garnovskii *et al.* (1993 ▶); Musie *et al.* (2001 ▶); Paul *et al.* (2002 ▶); Shi *et al.* (2007 ▶). For Schiff bases and biological systems, see: Anderson *et al.* (1997 ▶). For bond-length data, see: Allen *et al.* (1987 ▶).
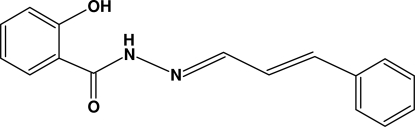


## Experimental

### 

#### Crystal data

C_16_H_14_N_2_O_2_
*M* *_r_* = 266.29
Monoclinic, 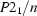

*a* = 4.8892 (6) Å
*b* = 26.563 (3) Å
*c* = 10.7367 (13) Å
β = 102.305 (2)°
*V* = 1362.4 (3) Å^3^
*Z* = 4
Mo *K*α radiation
μ = 0.09 mm^−1^
*T* = 295 K
0.15 × 0.12 × 0.10 mm

#### Data collection

Bruker SMART CCD area-detector diffractometer
Absorption correction: multi-scan (*SADABS*; Sheldrick, 1996 ▶) *T*_min_ = 0.987, *T*_max_ = 0.991
7141 measured reflections
2395 independent reflections
1354 reflections with *I* > 2σ(*I*)
*R*_int_ = 0.038

#### Refinement

*R*[*F* ^2^ > 2σ(*F* ^2^)] = 0.047
*wR*(*F* ^2^) = 0.123
*S* = 1.05
2395 reflections
183 parameters
H-atom parameters constrained
Δρ_max_ = 0.14 e Å^−3^
Δρ_min_ = −0.13 e Å^−3^

### 

Data collection: *SMART* (Siemens, 1996 ▶); cell refinement: *SAINT* (Siemens, 1996 ▶); data reduction: *SAINT*; program(s) used to solve structure: *SHELXS97* (Sheldrick, 2008 ▶); program(s) used to refine structure: *SHELXL97* (Sheldrick, 2008 ▶); molecular graphics: *SHELXTL* (Sheldrick, 2008 ▶); software used to prepare material for publication: *SHELXTL*.

## Supplementary Material

Crystal structure: contains datablocks global, I. DOI: 10.1107/S1600536808028481/cv2440sup1.cif

Structure factors: contains datablocks I. DOI: 10.1107/S1600536808028481/cv2440Isup2.hkl

Additional supplementary materials:  crystallographic information; 3D view; checkCIF report

## Figures and Tables

**Table 1:** Hydrogen-bond geometry (Å, °)

*D*—H⋯*A*	*D*—H	H⋯*A*	*D*⋯*A*	*D*—H⋯*A*
N1—H1*A*⋯O1	0.86	1.97	2.6348 (19)	133
O1—H1⋯O2^i^	0.82	2.10	2.804 (3)	144

Symmetry code: (i) 
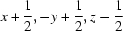
.

**Fig. 1.**
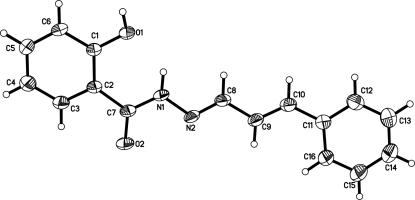
The molecular structure of (I), with displacement ellipsoids drawn at the 50% probability level.

## supplementary crystallographic information

### Comment

In recent years, a number of Schiff-bases have been investigated in terms of their coordination chemistry (Garnovskii *et al.*, 1993; Musie *et al.*, 2001; Paul *et al.*, 2002; Shi *et al.*, 2007;) and biological systems (Anderson *et al.*, 1997). In order to search for new Schiff-bases with higher bioactivity, the title compound, (I), was synthesized and its crystal structure determined.

In (I) (Fig. 1), the bond lengths and angles are in good agreement with the expected values (Allen *et al.*, 1987). The intramolecular N—H···O hydrogen bond (Table 1) influences the molecular conformation. In the crystal, the molecules are linked into infinite chains along direction [10-1] by O—H···O hydrogen bonds (Table 1).

### Experimental

The title compound was synthesized by the reaction of 2-Hydroxy-benzoic acid hydrazide(1 mmol, 152.2 mg) with 3-Phenyl-propenal (1 mmol, 132.2 mg) in ethanol(20 ml) under reflux conditions (348 K) for 6 h. The solvent was removed and the solid product recrystallized from tetrahydrofuran. After six days colorless crystals suitable for X-ray diffraction study were obtained. Yield, 226.3 mg, 85%. m.p. 239–241 K. Analysis calculated for C_16_H_14_N_2_O_2_: C 72.16, H 5.30, N 10.52%; found: C 71.73, H 5.34, N 10.48%.

### Refinement

All H atoms were placed in idealized positions (C—H = 0.93— 0.97 Å, N—H = 0.86 Å) and refined as riding, with *U*_iso_(H) = 1.2 or 1.5*U*_eq_(C, N).

### Crystal data

**Table:**

C_16_H_14_N_2_O_2_	*F*(000) = 560
*M**_r_* = 266.29	*D*_x_ = 1.298 Mg m^−^^3^
Monoclinic, *P*2_1_/*n*	Mo *K*α radiation, λ = 0.71073 Å
Hall symbol: -P 2yn	Cell parameters from 932 reflections
*a* = 4.8892 (6) Å	θ = 3.6–21.4°
*b* = 26.563 (3) Å	µ = 0.09 mm^−^^1^
*c* = 10.7367 (13) Å	*T* = 295 K
β = 102.305 (2)°	Block, colourless
*V* = 1362.4 (3) Å^3^	0.15 × 0.12 × 0.10 mm
*Z* = 4	

### Data collection

**Table:**

Bruker SMART CCD area-detector diffractometer	2395 independent reflections
Radiation source: fine-focus sealed tube	1354 reflections with *I* > 2σ(*I*)
graphite	*R*_int_ = 0.038
φ and ω scans	θ_max_ = 25.1°, θ_min_ = 2.5°
Absorption correction: multi-scan (SADABS; Sheldrick, 1996)	*h* = −5→5
*T*_min_ = 0.987, *T*_max_ = 0.991	*k* = −21→31
7141 measured reflections	*l* = −12→12

### Refinement

**Table:**

Refinement on *F*^2^	Secondary atom site location: difference Fourier map
Least-squares matrix: full	Hydrogen site location: inferred from neighbouring sites
*R*[*F*^2^ > 2σ(*F*^2^)] = 0.047	H-atom parameters constrained
*wR*(*F*^2^) = 0.124	*w* = 1/[σ^2^(*F*_o_^2^) + (0.0505*P*)^2^] where *P* = (*F*_o_^2^ + 2*F*_c_^2^)/3
*S* = 1.05	(Δ/σ)_max_ < 0.001
2395 reflections	Δρ_max_ = 0.14 e Å^−^^3^
183 parameters	Δρ_min_ = −0.13 e Å^−^^3^
0 restraints	Extinction correction: SHELXL97 (Sheldrick, 1997a), Fc^*^=kFc[1+0.001xFc^2^λ^3^/sin(2θ)]^-1/4^
Primary atom site location: structure-invariant direct methods	Extinction coefficient: 0.008 (2)

### Special details

**Table:**

Geometry. All e.s.d.'s (except the e.s.d. in the dihedral angle between two l.s. planes) are estimated using the full covariance matrix. The cell e.s.d.'s are taken into account individually in the estimation of e.s.d.'s in distances, angles and torsion angles; correlations between e.s.d.'s in cell parameters are only used when they are defined by crystal symmetry. An approximate (isotropic) treatment of cell e.s.d.'s is used for estimating e.s.d.'s involving l.s. planes.
Refinement. Refinement of *F*^2^ against ALL reflections. The weighted *R*-factor *wR* and goodness of fit *S* are based on *F*^2^, conventional *R*-factors *R* are based on *F*, with *F* set to zero for negative *F*^2^. The threshold expression of *F*^2^ > σ(*F*^2^) is used only for calculating *R*-factors(gt) *etc*. and is not relevant to the choice of reflections for refinement. *R*-factors based on *F*^2^ are statistically about twice as large as those based on *F*, and *R*- factors based on ALL data will be even larger.

### Fractional atomic coordinates and isotropic or equivalent isotropic displacement parameters (Å^2^)

**Table:**

	*x*	*y*	*z*	*U*_iso_*/*U*_eq_	
O1	0.5440 (4)	0.26610 (6)	0.08347 (14)	0.0702 (5)	
H1	0.6201	0.2633	0.0227	0.105*	
O2	0.4213 (4)	0.27755 (6)	0.45173 (13)	0.0687 (5)	
N1	0.2766 (4)	0.24015 (6)	0.26236 (15)	0.0508 (5)	
H1A	0.2861	0.2378	0.1835	0.061*	
N2	0.1041 (4)	0.20892 (7)	0.31262 (16)	0.0513 (5)	
C1	0.6656 (4)	0.30391 (8)	0.15959 (19)	0.0483 (6)	
C2	0.6118 (4)	0.30864 (8)	0.28157 (18)	0.0450 (5)	
C3	0.7379 (5)	0.34777 (9)	0.3569 (2)	0.0634 (7)	
H3	0.7048	0.3513	0.4386	0.076*	
C4	0.9102 (6)	0.38164 (9)	0.3154 (2)	0.0736 (8)	
H4	0.9898	0.4080	0.3676	0.088*	
C5	0.9638 (5)	0.37611 (9)	0.1959 (2)	0.0662 (7)	
H5	1.0826	0.3986	0.1674	0.079*	
C6	0.8438 (5)	0.33774 (9)	0.1184 (2)	0.0606 (7)	
H6	0.8818	0.3343	0.0375	0.073*	
C7	0.4304 (5)	0.27445 (8)	0.33872 (19)	0.0476 (6)	
C8	−0.0380 (5)	0.17709 (8)	0.2358 (2)	0.0529 (6)	
H8	−0.0220	0.1766	0.1510	0.064*	
C9	−0.2215 (5)	0.14220 (8)	0.2788 (2)	0.0530 (6)	
H9	−0.2416	0.1442	0.3628	0.064*	
C10	−0.3630 (5)	0.10743 (9)	0.2044 (2)	0.0576 (6)	
H10	−0.3419	0.1072	0.1203	0.069*	
C11	−0.5490 (5)	0.06929 (8)	0.2383 (2)	0.0529 (6)	
C12	−0.6805 (5)	0.03509 (10)	0.1480 (2)	0.0739 (8)	
H12	−0.6499	0.0369	0.0655	0.089*	
C13	−0.8563 (6)	−0.00161 (11)	0.1777 (3)	0.0842 (9)	
H13	−0.9404	−0.0244	0.1156	0.101*	
C14	−0.9071 (6)	−0.00457 (10)	0.2973 (3)	0.0761 (8)	
H14	−1.0287	−0.0288	0.3169	0.091*	
C15	−0.7772 (6)	0.02847 (10)	0.3879 (3)	0.0807 (8)	
H15	−0.8084	0.0264	0.4702	0.097*	
C16	−0.6002 (5)	0.06496 (9)	0.3590 (2)	0.0670 (7)	
H16	−0.5137	0.0871	0.4223	0.080*	

### Atomic displacement parameters (Å^2^)

**Table:**

	*U*^11^	*U*^22^	*U*^33^	*U*^12^	*U*^13^	*U*^23^
O1	0.0858 (13)	0.0893 (13)	0.0482 (9)	−0.0339 (10)	0.0428 (9)	−0.0229 (9)
O2	0.0900 (14)	0.0857 (12)	0.0392 (9)	−0.0137 (9)	0.0337 (8)	−0.0060 (8)
N1	0.0615 (13)	0.0615 (12)	0.0363 (9)	−0.0068 (10)	0.0257 (9)	0.0017 (9)
N2	0.0587 (13)	0.0596 (12)	0.0424 (10)	−0.0030 (10)	0.0263 (9)	0.0069 (9)
C1	0.0531 (15)	0.0568 (14)	0.0384 (12)	−0.0055 (11)	0.0174 (10)	−0.0034 (10)
C2	0.0509 (15)	0.0505 (13)	0.0366 (11)	0.0016 (11)	0.0158 (10)	0.0003 (10)
C3	0.083 (2)	0.0703 (17)	0.0416 (13)	−0.0132 (14)	0.0247 (12)	−0.0088 (12)
C4	0.097 (2)	0.0700 (18)	0.0568 (16)	−0.0233 (15)	0.0219 (15)	−0.0121 (13)
C5	0.0740 (19)	0.0696 (17)	0.0575 (15)	−0.0225 (14)	0.0192 (14)	0.0020 (13)
C6	0.0701 (18)	0.0765 (17)	0.0412 (12)	−0.0134 (13)	0.0252 (12)	0.0026 (12)
C7	0.0552 (15)	0.0547 (14)	0.0383 (12)	0.0047 (11)	0.0218 (11)	0.0002 (11)
C8	0.0614 (17)	0.0631 (15)	0.0377 (12)	−0.0015 (12)	0.0183 (11)	0.0042 (11)
C9	0.0580 (16)	0.0613 (15)	0.0440 (13)	−0.0030 (12)	0.0205 (11)	0.0068 (11)
C10	0.0613 (17)	0.0687 (16)	0.0444 (13)	−0.0007 (13)	0.0151 (12)	0.0044 (12)
C11	0.0528 (16)	0.0563 (15)	0.0501 (14)	0.0024 (12)	0.0118 (11)	0.0029 (12)
C12	0.079 (2)	0.086 (2)	0.0571 (16)	−0.0149 (16)	0.0147 (14)	−0.0071 (14)
C13	0.082 (2)	0.082 (2)	0.085 (2)	−0.0215 (16)	0.0086 (17)	−0.0090 (16)
C14	0.0696 (19)	0.0665 (19)	0.093 (2)	−0.0088 (14)	0.0189 (16)	0.0139 (16)
C15	0.092 (2)	0.082 (2)	0.0768 (18)	−0.0177 (16)	0.0376 (17)	0.0056 (16)
C16	0.076 (2)	0.0684 (17)	0.0609 (16)	−0.0142 (13)	0.0244 (14)	−0.0028 (12)

### Geometric parameters (Å, °)

**Table:**

O1—C1	1.350 (2)	C8—C9	1.433 (3)
O1—H1	0.8200	C8—H8	0.9300
O2—C7	1.226 (2)	C9—C10	1.317 (3)
N1—C7	1.344 (2)	C9—H9	0.9300
N1—N2	1.373 (2)	C10—C11	1.458 (3)
N1—H1A	0.8600	C10—H10	0.9300
N2—C8	1.278 (2)	C11—C16	1.376 (3)
C1—C6	1.388 (3)	C11—C12	1.383 (3)
C1—C2	1.395 (3)	C12—C13	1.381 (3)
C2—C3	1.379 (3)	C12—H12	0.9300
C2—C7	1.490 (3)	C13—C14	1.361 (3)
C3—C4	1.370 (3)	C13—H13	0.9300
C3—H3	0.9300	C14—C15	1.362 (3)
C4—C5	1.371 (3)	C14—H14	0.9300
C4—H4	0.9300	C15—C16	1.378 (3)
C5—C6	1.366 (3)	C15—H15	0.9300
C5—H5	0.9300	C16—H16	0.9300
C6—H6	0.9300		
			
C1—O1—H1	109.5	N2—C8—H8	119.6
C7—N1—N2	118.73 (17)	C9—C8—H8	119.6
C7—N1—H1A	120.6	C10—C9—C8	122.9 (2)
N2—N1—H1A	120.6	C10—C9—H9	118.6
C8—N2—N1	116.17 (17)	C8—C9—H9	118.6
O1—C1—C6	120.93 (18)	C9—C10—C11	127.6 (2)
O1—C1—C2	119.23 (18)	C9—C10—H10	116.2
C6—C1—C2	119.8 (2)	C11—C10—H10	116.2
C3—C2—C1	117.9 (2)	C16—C11—C12	117.0 (2)
C3—C2—C7	116.66 (18)	C16—C11—C10	122.8 (2)
C1—C2—C7	125.41 (19)	C12—C11—C10	120.2 (2)
C4—C3—C2	122.2 (2)	C13—C12—C11	121.4 (2)
C4—C3—H3	118.9	C13—C12—H12	119.3
C2—C3—H3	118.9	C11—C12—H12	119.3
C3—C4—C5	119.2 (2)	C14—C13—C12	120.4 (3)
C3—C4—H4	120.4	C14—C13—H13	119.8
C5—C4—H4	120.4	C12—C13—H13	119.8
C6—C5—C4	120.5 (2)	C13—C14—C15	119.0 (3)
C6—C5—H5	119.8	C13—C14—H14	120.5
C4—C5—H5	119.8	C15—C14—H14	120.5
C5—C6—C1	120.4 (2)	C14—C15—C16	120.8 (3)
C5—C6—H6	119.8	C14—C15—H15	119.6
C1—C6—H6	119.8	C16—C15—H15	119.6
O2—C7—N1	121.0 (2)	C11—C16—C15	121.3 (2)
O2—C7—C2	121.1 (2)	C11—C16—H16	119.3
N1—C7—C2	117.84 (17)	C15—C16—H16	119.3
N2—C8—C9	120.8 (2)		

### Hydrogen-bond geometry (Å, °)

**Table:**

*D*—H···*A*	*D*—H	H···*A*	*D*···*A*	*D*—H···*A*
N1—H1A···O1	0.86	1.97	2.6348 (19)	133.
O1—H1···O2^i^	0.82	2.10	2.804 (3)	144.
O1—H1···N2^i^	0.82	2.36	3.057 (3)	144.

Symmetry codes: (i) *x*+1/2, −*y*+1/2, *z*−1/2.
